# Characterisation of CD154^+^ T cells following *ex vivo* allergen stimulation illustrates distinct T cell responses to seasonal and perennial allergens in allergic and non-allergic individuals

**DOI:** 10.1186/1471-2172-14-49

**Published:** 2013-11-05

**Authors:** Karen A Smith, Nicola J Gray, Femi Saleh, Elizabeth Cheek, Anthony J Frew, Florian Kern, Michael D Tarzi

**Affiliations:** 1 Brighton and Sussex Medical School, division of clinical medicine, pathogen-host-interactions group, University of Sussex, Brighton BN1 9PS, UK; 2 Department of Respiratory Medicine, Royal Sussex County Hospital, Brighton BN2 5BE, UK; 3 Department of Computing, Engineering and Mathematics, University of Brighton, Brighton BN2 4GJ, UK; 4 Brighton and Sussex Medical School, University of Sussex, Brighton BN1 9PX, UK

**Keywords:** Allergen-specific T cell, Cat dander allergy, CD154, Grass pollen allergy, *ex vivo* phenotyping, Flow cytometry

## Abstract

**Background:**

Allergic sensitisation has been ascribed to a dysregulated relationship between allergen-specific Th1, Th2 and regulatory T cells. We sought to utilise our short-term CD154 detection method to further analyse the relationship between these T cell subsets and investigate differences between seasonal and perennial allergens. Using peripheral blood samples from grass-allergic, cat-allergic and healthy non-atopic subjects, we compared the frequencies and phenotype of CD154-positive T helper cells following stimulation with seasonal (grass) and perennial (cat dander) allergens.

**Results:**

We identified a higher frequency of CD154^+^ T cells in grass-allergic individuals compared to healthy controls; this difference was not evident following stimulation with cat allergen. Activated Th1, Th2 and Tr1-like cells, that co-express IFNγ, IL4 and IL10, respectively, were identified in varying proportions in grass-allergic, cat-allergic and non-allergic individuals. We confirmed a close correlation between Th1, Th2 and Tr1-like cell frequency in non-allergic volunteers, such that the three parameters increased together to maintain a low Th2: Th1 ratio. This relationship was dysregulated in grass-allergic individuals with no correlation between the T cell subsets and a higher Th2: Th1 ratio. We confirmed previous reports of a late-differentiated T cell phenotype in response to seasonal allergens compared to early-differentiated T cell responses to perennial allergens.

**Conclusions:**

The findings confirm our existing work illustrating an important balance between Th1, Th2 and Tr1-like responses to allergens in health, where Th2 responses are frequently observed, but balanced by Th1 and regulatory responses. We confirm previous tetramer-based reports of phenotypic differences in T cells responding to seasonal and perennial allergens.

## Background

The early activation marker CD154 is transiently expressed following ligation of the T cell receptor, providing direct access to an antigen-specific population following *ex vivo* stimulation [1,2]. Whereas MHC tetramers define responses to immunodominant epitopes in subjects with the appropriate HLA-DR haplotype, the CD154 method permits direct access to a larger group of responding T cells that can be simultaneously characterised by cytokine expression. We recently presented the first detailed description of CD154^+^ T cells after short-term birch allergen stimulation in non-allergic controls and birch pollinosis patients [3]. Using this method, we showed that T cell responses to birch allergen in health are of mixed phenotype, but tightly maintained at a low Th2: Th1 ratio. By contrast, the relationship was dysregulated in birch pollinosis, with a higher Th2: Th1 ratio and abrogation of the IL-10 response.

Experiments using MHC class II tetramer technology have described important differences between T cell responses to seasonal and perennial allergens. In particular, T cells specific for seasonal allergens have been reported as CD27^low^, late-differentiated cells [4-6], whereas perennial allergen-specific T cell responses are CD27^high^, consistent with a central memory/early differentiated phenotype [7,8]. Our previous study did demonstrate a significantly higher frequency of CD27^-^ late-differentiated T cells responding to birch pollen in allergic individuals compared to non-atopic controls [3]. However, the difference was not striking, possibly because the birch extract antigen used in the system activates T cells that are specific for other PR-10 protein homologs found in plant derived foods.

The objectives of this research were [1] to explore whether the relationship between Th1 and Th2 cells previously defined using birch allergen also hold for cat and grass allergens, and [2] to investigate whether previously described differences between T cell responses to a seasonal (grass pollen) and perennial (cat dander) allergen could be reproduced using the CD154 detection method.

## Results

### The responder cell frequency differs according to the allergen and atopic status

**Figure 1:**
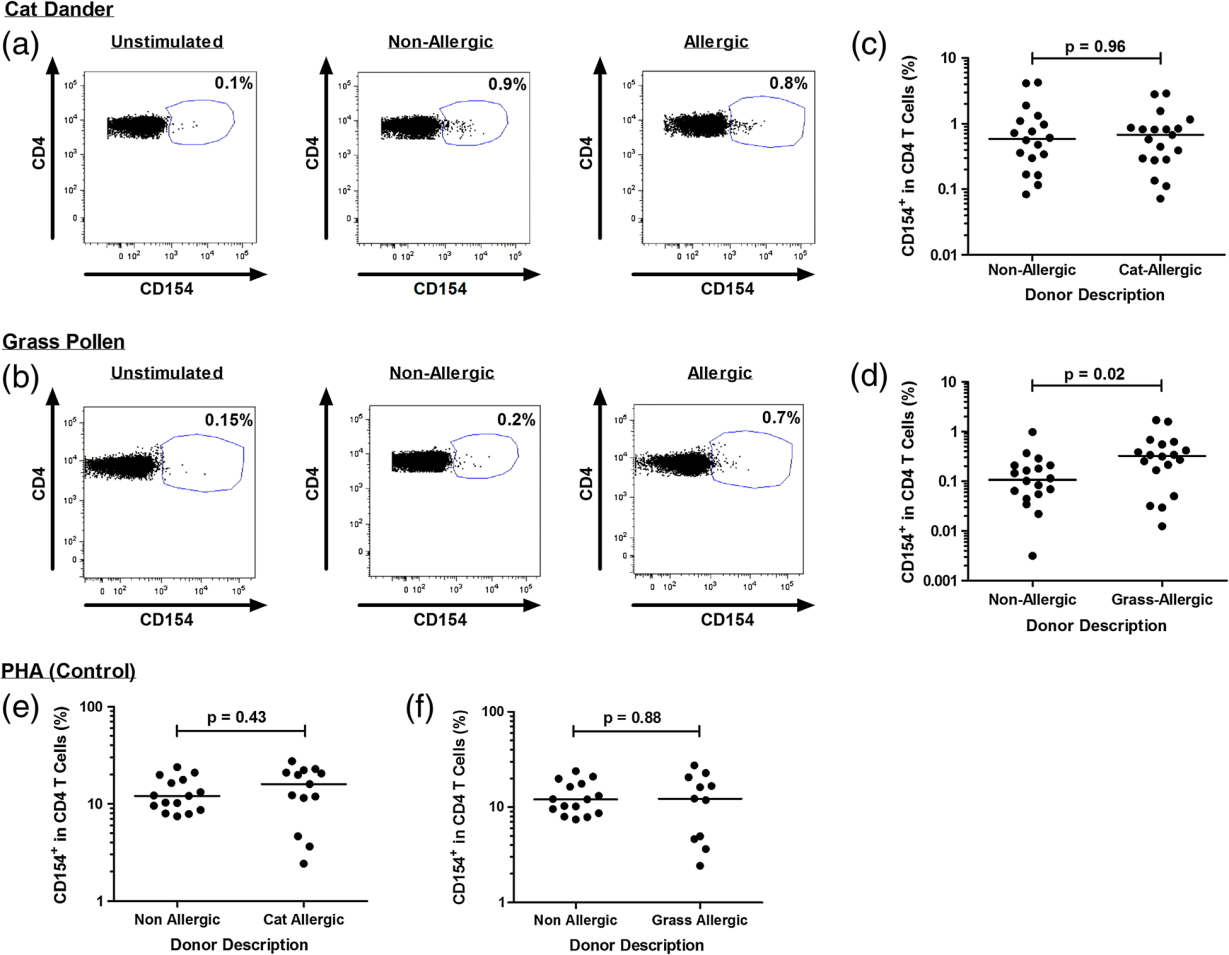
**Cat and grass allergen-induced CD154 expression in allergic and non-allergic individuals.** Flow cytometric dot plots illustrate CD4^+^ CD154 expression in allergic and non-allergic individuals in **(a, c, e, g)** unstimulated T-cells and following PBMC stimulation with **(b, d)** cat dander and **(f, h)** grass pollen allergen extracts. Scatter dot plots demonstrate the background-corrected percentage of **(c)** cat allergen-specific and **(d)** grass allergen-specific CD154^+^ T cells in allergic and non-allergic individuals following allergen stimulation. PHA-induced CD154^+^ T cell population in non-allergic, **(e)** cat-allergic and **(f)** grass-allergic individuals.

A CD4^+^CD154^+^ T cell population was resolved in all study participants following cat and grass allergen stimulation (Figure 1a, b). In additional experiments, PBMC were stimulated under the same conditions with 1.54 ng/ml LPS (representing the LPS content of the allergen extracts) or PBS: this did not induce CD4 T cell CD154 expression over background (data not shown).

The cat allergen-induced CD154^+^ T cell response represented 0.58% and 0.74% of the total CD4 T cell population in non-allergic and cat-allergic individuals, respectively. The difference did not reach statistical significance (p = 0.96, Figure 1c).

The frequency of CD154^+^ T cells responding to grass pollen allergen was lower compared to cat allergen, representing 0.11% and 0.33% of all CD4 T cells in non-allergic and grass-allergic individuals, respectively (Figure 1d). Grass-allergic individuals exhibited a significantly higher frequency of CD154^+^ T cells compared to non-allergic controls (p = 0.02, Figure 1d).

### Th1 and Th2 responses to both allergens are mixed with substantial overlap, but the Th2: Th1 ratio separates allergic and non-allergic individuals

**Figure 2:**
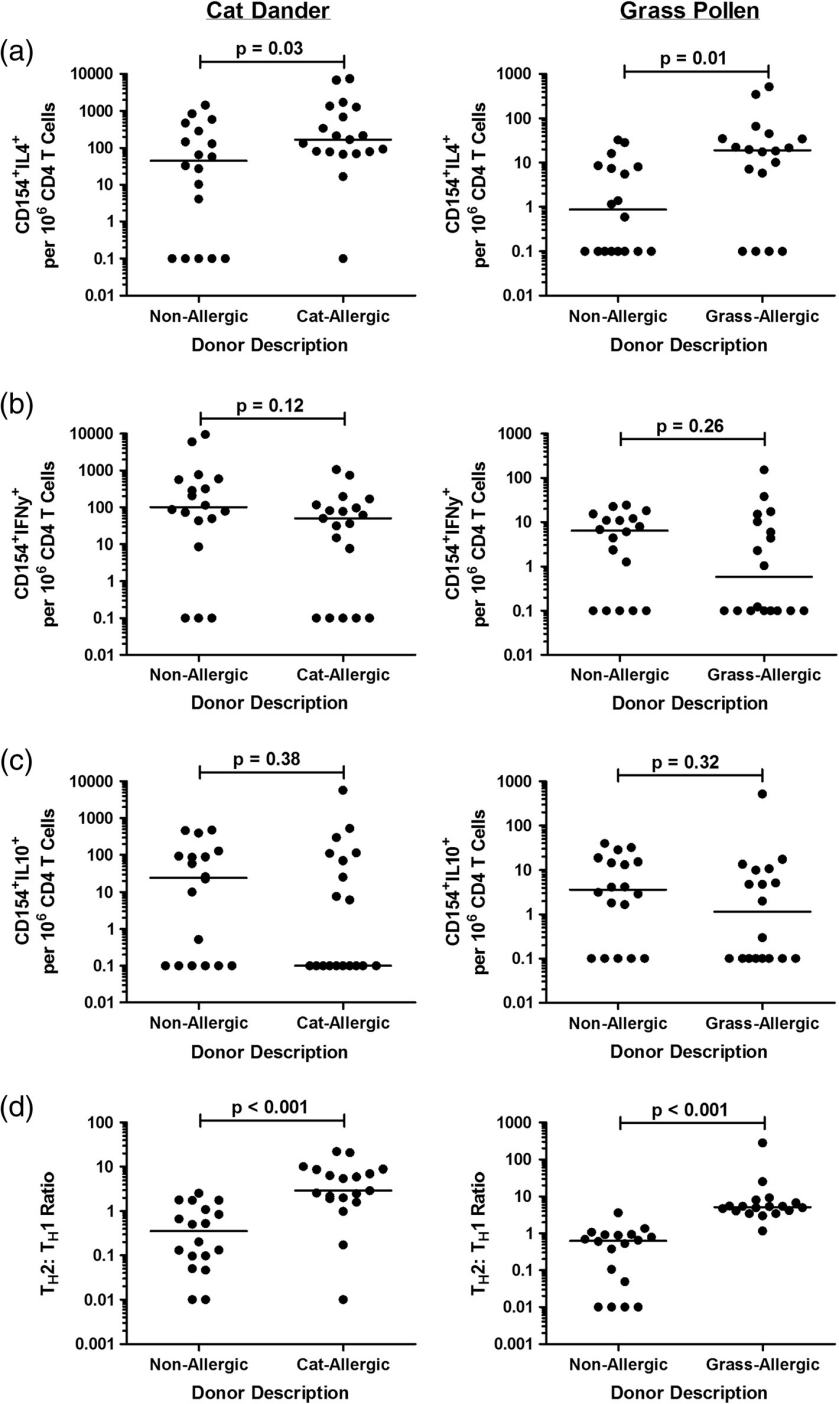
**CD154+ Th1 and Th2 cytokine populations following stimulation with cat and grass allergens in non-allergic and cat-allergic individuals.** Flow cytometric dot plots illustrate Th1 and Th2 cytokine expression within CD154^+^ T cell populations following PBMC stimulation with cat dander and grass pollen allergens in representative non-allergic and allergic individuals.

**Figure 3:**
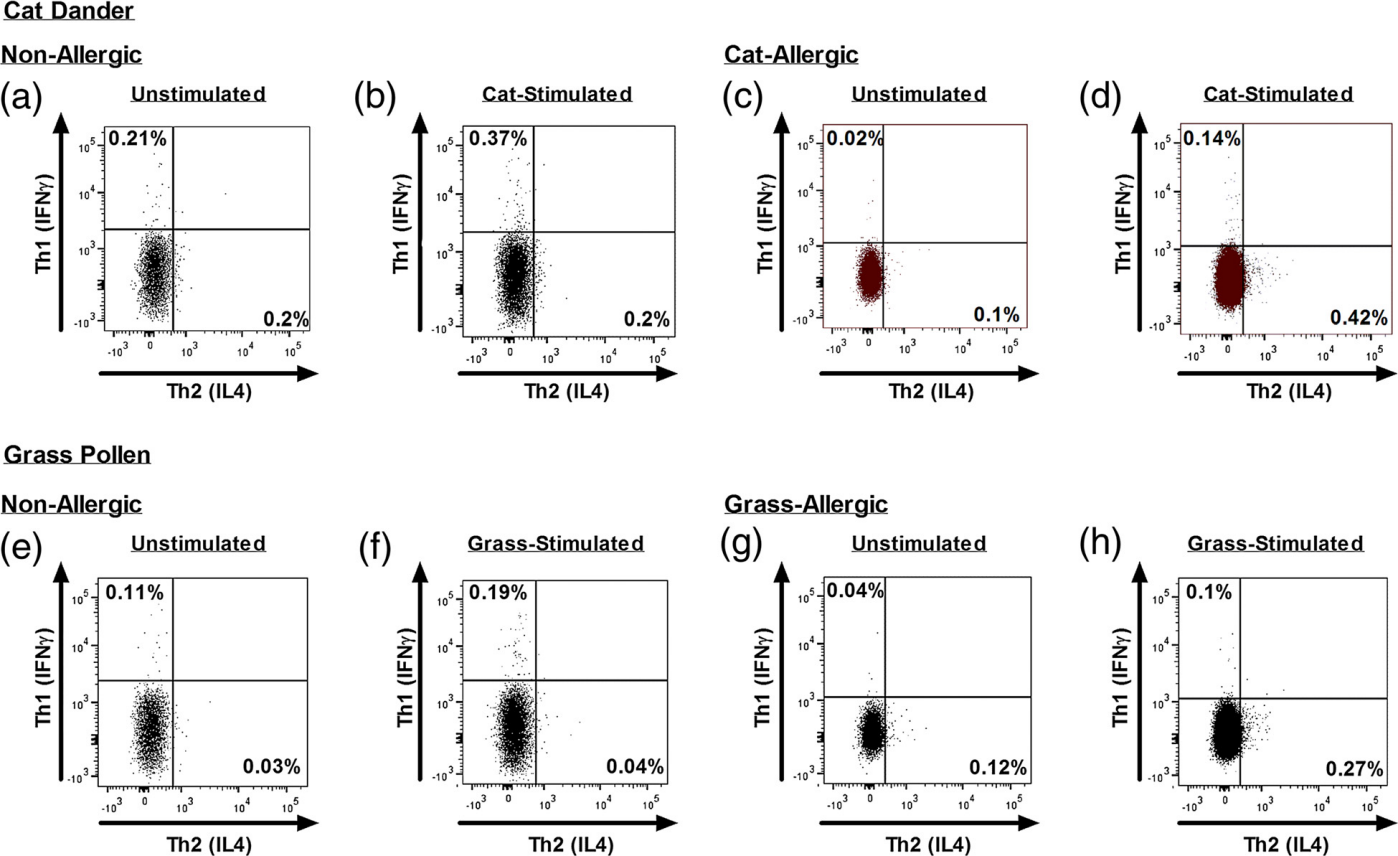
**Allergen-induced Th1, Th2 and Tr1-like cell populations in allergic and non-allergic participants.** Data represents **(a)** CD154^+^IL-4^+^ Th2 cells, **(b)** CD154^+^IFNγ^+^ Th1 cells and **(c)** CD154^+^IL-10^+^ Tr1-like cells illustrated as the background-corrected frequency of positive cells per 10^6^ CD4 T cells on a log scale, **(d)** Th2: Th1 ratio.

Th1, Th2 and Tr1-like cells were defined as CD154^+^IFNγ^+^, CD154^+^IL-4^+^ and CD154^+^IL-10^+^, respectively (Figure 2). With respect to both cat and grass allergens, we observed very mixed and variable T cell responses in both allergic and non-allergic subjects. In the case of grass allergen, Th2 responses were convincingly greater than the non-allergic group (p = 0.01), but the parameter separated the cat-allergic and cat-tolerant group less completely (p = 0.03) (Figure 3a). There were no differences in the frequency of Th1 or Tr1-like responses between allergic and non-allergic participants in response to either cat or grass allergens (Figure 3b, c).

However, the Th2: Th1 ratio (calculated by dividing the frequency of CD154^+^IL-4^+^ Th2 cells by the frequency of CD154^+^IFNγ^+^ Th1 cells) achieved better separation of cat and grass-allergic participants from non-allergic subjects (Figure 3d).

T cell responses to the control mitogen PHA did not differ between allergic and non-allergic individuals (Figure 1e-f).

### A close correlation between Th1, Th2 and Tr1-like responses in healthy subjects is dysregulated in allergic individuals

To explore dependency between Th2 responses to allergens and other parameters, we correlated Th2 cell frequency with serological and immunological responses.

**Figure 4:**
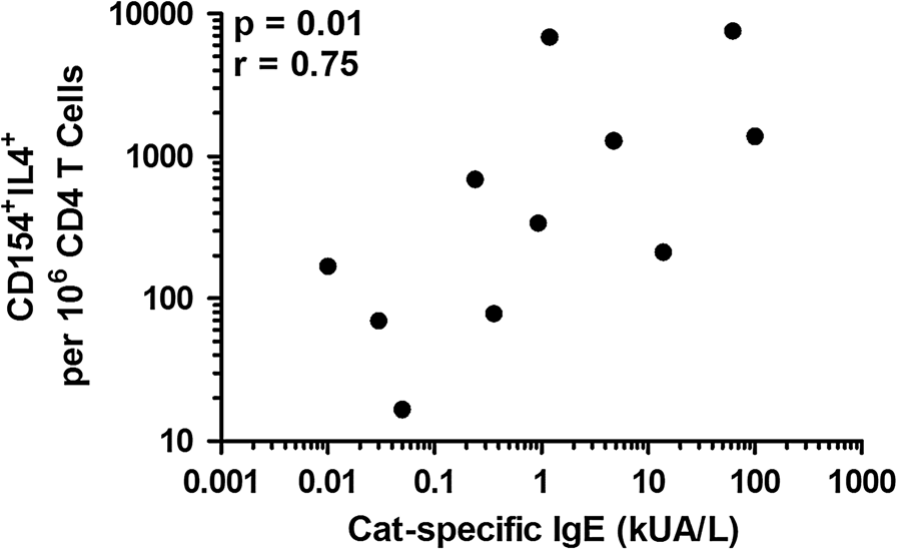
**Correlation of CD154**^**+**^**IL-4**^**+ **^**cell frequency and IgE levels in cat-allergic individuals.**

A close correlation was observed between Th2 cell frequency and the absolute concentration of cat-specific IgE (analysis restricted to eleven of 19 cat-allergic participants (p = 0.01, r = 0.75, Figure 4).

**Figure 5:**
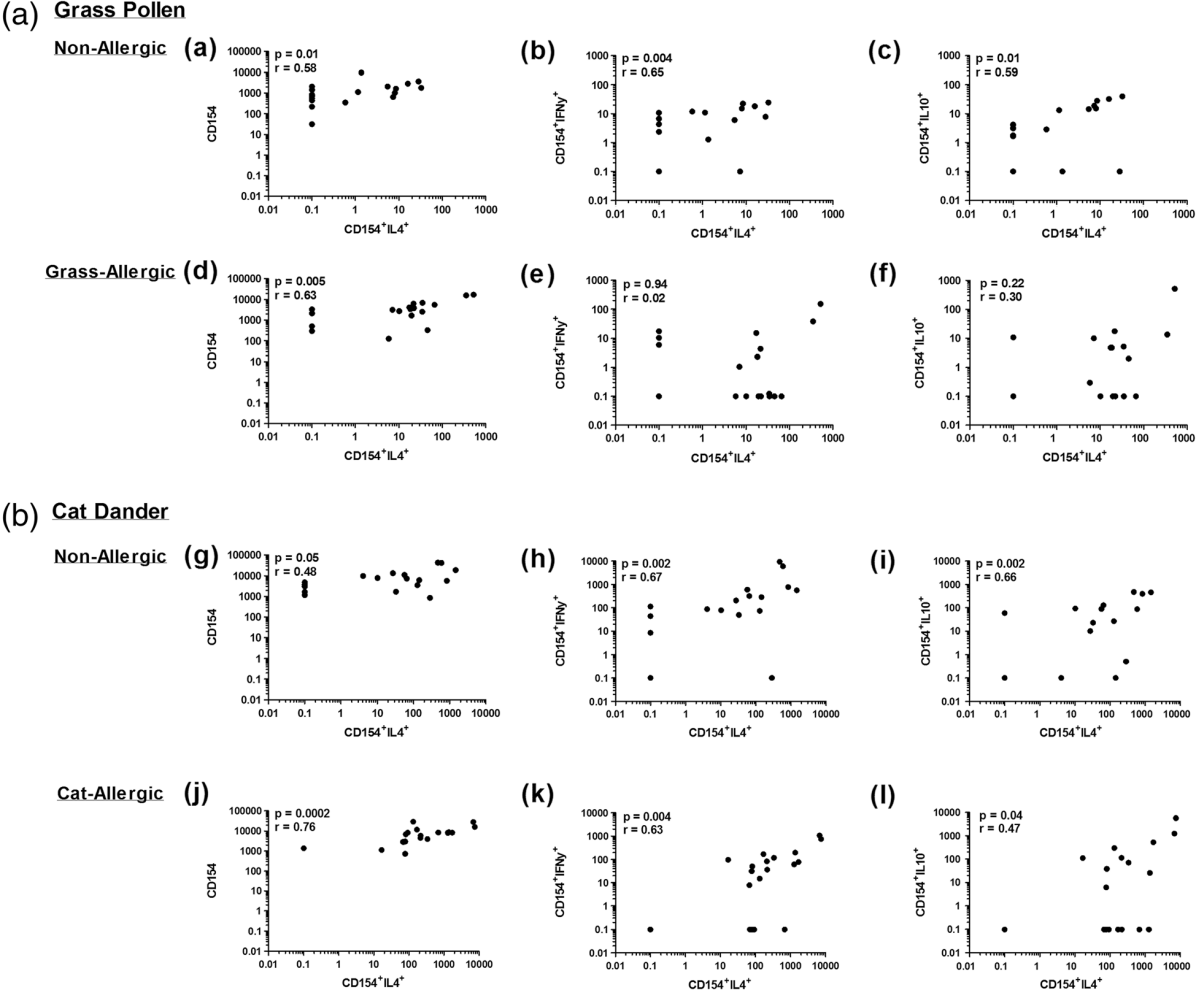
**Correlations between Th2, Th1 and Tr1-like cell populations in allergic and non-allergic individuals following (a) cat allergen and (b) grass allergen stimulation.** Data represents the background-corrected frequency of CD154^+^IL-4^+^ Th2 cells, CD154^+^IFNγ^+^ Th1 cells and CD154^+^IL-10^+^ Tr1-like cells per 10^6^ CD4 T cells.

Amongst non-atopic participants, positive correlations were observed between the frequency of Th2 cells and the Th1 and Tr1-like responses following PBMC stimulation with either cat or grass allergen (p < 0.01, Figure 5a-c, g-i), such that the expression of these cytokines increased together to maintain a low Th2: Th1 ratio.

Amongst grass-allergic participants, there was no dependence between the frequency of Th2, Th1 and Tr1-like cells (Figure 5d-f). In cat-allergic participants, positive correlations were noted between the frequency of Th2 cells and the Th1 (p = 0.004, Figure 5k) and the Tr1-like (p = 0.04, Figure 5l) response, however at a lower significance compared to their non-allergic counterparts and at a higher Th2: Th1 ratio.

### Th2 responses to grass, but not cat allergen, have a late-differentiated phenotype

**Figure 6:**
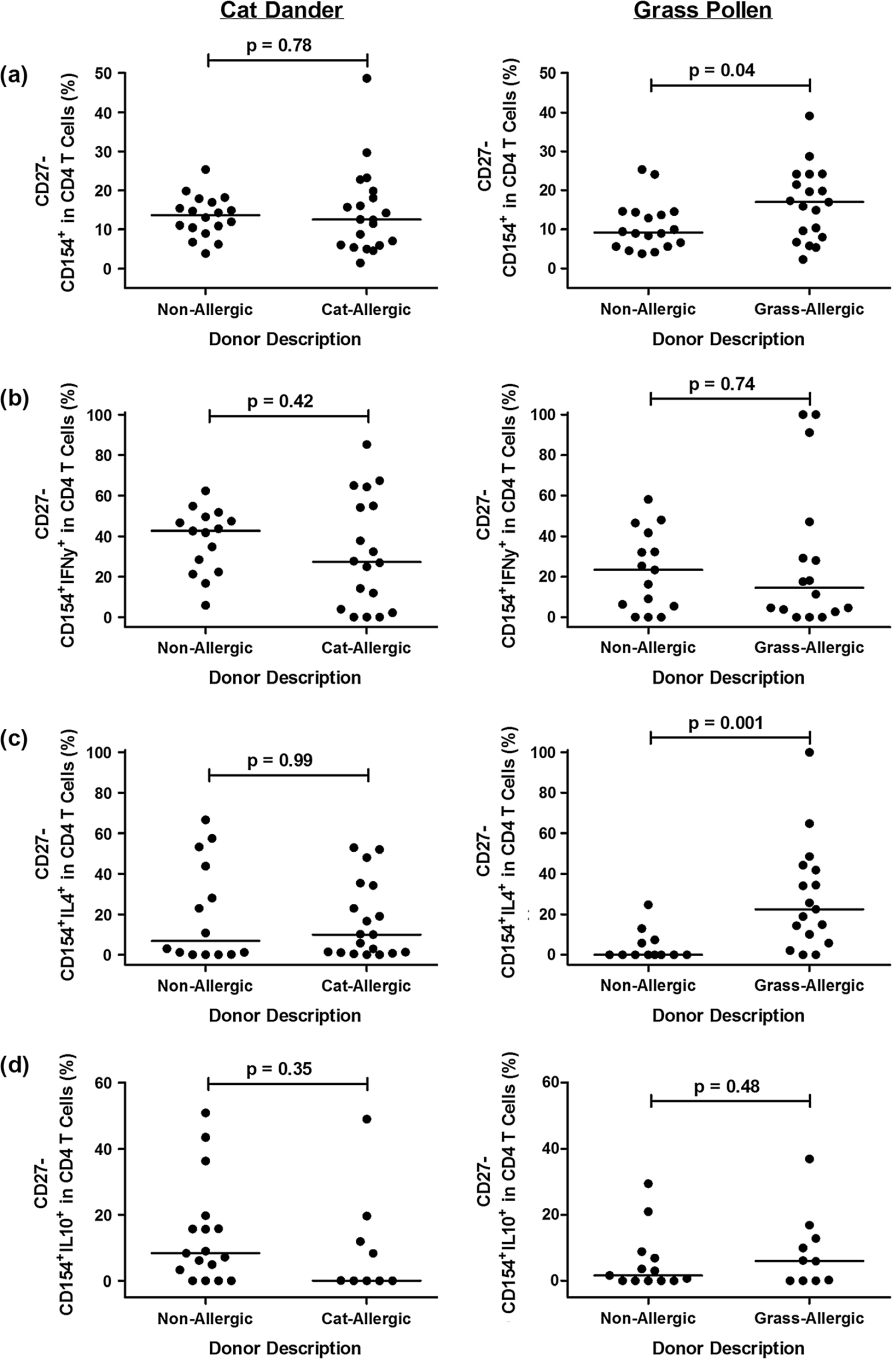
**Phenotypic analysis of allergen-stimulated T cell populations based on CD27 expression.** Data represents the phenotype of **(a)** CD154, **(b)** CD154^+^IFNγ^+^, **(c)** CD154^+^IL-4^+^ and **(d)** CD154^+^ IL-10^+^ T cells responses in non-allergic, cat-allergic and grass-allergic individuals, expressed as the background-corrected percentage of positive cells of the total responding CD4 T cell population. CD154^+^ and CD154^+^cytokine^+^ T cell populations were initially calculated using Boolean gating and subsequently analysed for CD27 expression.

We investigated the phenotype of responding CD154^+^ and CD154^+^cytokine^+^ T cell populations following allergen stimulation using the expression of cell surface marker CD27. Subsets were defined as early-differentiated (CD27^+^) or late-differentiated (CD27^-^). T cell responses to cat allergen were predominantly early-differentiated CD27^-^ cells, regardless of atopic status (Figure 6a-d). In patients with grass pollinosis, the proportion of late-differentiated CD27^-^ Th2 cells was far higher in the allergic group compared to the non-allergic group (Figure 6c); there was a slight increase in the proportion of CD27^-^ cells in the total (CD154^+^) response, but no difference with respect to Th1 responses.

### Subjects with asymptomatic sensitisation and those with allergic disease cannot be differentiated on the basis of the Th2: Th1 ratio

**Figure 7:**
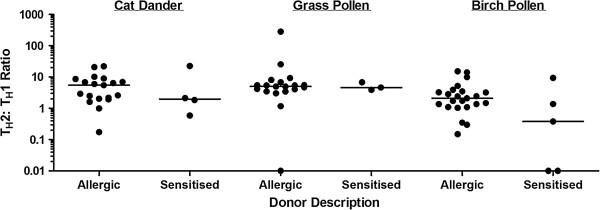
Comparison of the Th2: Th1 ratio following birch, cat and grass allergen stimulation in allergic and sensitised individuals.

We also investigated the T cell response to allergens in a small group of individuals with asymptomatic sensitisation to birch, cat or grass allergen, defined as a positive skin prick test to the allergen but no clinical symptoms upon exposure to cats or during the relevant pollen season. Following stimulation with the appropriate allergen, the Th2: Th1 ratio in sensitised individuals was similar to that of allergic subjects, demonstrating that this parameter does not discriminate between sensitisation and allergic disease (Figure 7).

## Discussion

CD154 expression has been demonstrated to identify an antigen-specific T cell population following short-term stimulation with pathogen-derived antigens [1,2]. The method is technically straightforward, is not HLA-restricted and is compatible with intracellular staining for cytokine expression, making it an attractive alternative to tetramer staining in some situations. We recently presented the first detailed description of CD154^+^ T cell responses following birch allergen stimulation in allergic and non-allergic subjects [3]. In this study, we explore the frequency and phenotype of T cells responding to grass pollen – a true seasonal allergen – and cat allergen, a perennial allergen. The findings are confirmatory of previous reports, and include: a higher frequency of T cells responding to cat dander compared to grass pollen allergen, a higher frequency of late-differentiated CD27- T cells responding to grass pollen compared to cat dander allergen; a mixed T cell responses to allergens, but with a higher Th2: Th1 ratio in sensitised subjects. We also demonstrate a close correlation between the frequency of Th2 cells responding to cat dander allergen and the cat allergen-specific IgE level and show that the assay system cannot differentiate between asymptomatic sensitisation and allergic disease.

The allergen-specificity of T cell responses in our experiments have not been confirmed, which is a weakness of the study. The CD154^+^ population in our experiments is far greater than the allergen-specific cell frequency reported using other methods [9], and most likely represents an enriched population. The vast majority of our positive findings relate to the CD154/cytokine double-positive population, the frequency of which is more in keeping with previous literature. Demonstrating allergen specificity in this assay system is not straightforward: intracellular staining for CD154 precludes live cell sorting; specificity for pathogen-reactive T cells has been demonstrated using a modified method that utilises extracellular CD154 staining [10], however, the frequency of responding cells following allergen stimulation is small, and cloning experiments – with attendant limitations – would be required. A previous study with CD154/tetramer co-staining after prolonged cell culture followed by re-stimulation questioned the validity of CD154 as a marker for allergen-specific T cells (ref). However, these conditions are known to invalidate the assay, which is only reliable after short-term stimulation [11].

Although the allergen-specificity of the T cell responses in this assay system have not been formally demonstrated, our previous paper highlights important supportive information: the frequency of Th2 cells responding to birch allergen correlates with the levels of birch-specific IgE, providing a direct link between a cellular parameter that purports to be antigen-specific and a serological parameter that is definitely allergen-specific; the Th2: Th1 ratio after PBMC stimulation with a control allergen to which an atopic subject was not sensitised was low; the frequency of T cell responding to birch allergen increased during the birch season [3]. Confirmation of a strong correlation between Th2 cells responding to cat dander and levels of cat-dander IgE in this paper provides further support, in addition to confirmation of phenotypic data established using MHC class II tetramers. In keeping with our previous studies of responses to birch allergen [3] and other studies [7,8,11], we found that responses to cat and grass allergens were very mixed, with activated Th2, Th1 and Tr1-like lymphocytes identified in varying proportions in allergic and non-allergic participants. Although the frequency of Th2 cells was significantly higher amongst allergic participants compared to non-allergic controls, there was considerable overlap, and the frequency of Th1 cells did not differ significantly. The overlap of Th2 responses to cat allergen was particularly marked; notably, a modified Th2 response to cat allergen in health has been reported by Platts-Mills *et al.*[12] characterised by strong IL-4 and IL-10 production, albeit in highly-exposed subjects.

Using correlation analysis, we demonstrate a novel relationship between the frequencies of T cell subsets in grass and cat allergen tolerance, maintaining a low Th2: Th1 ratio and an appropriate frequency of IL-10^+^ T cells. This relationship was dysregulated in allergic patients, in whom the correlation between T cell subsets was weaker or lost and the Th2: Th1 ratio maintained at higher levels. This mathematical relationship between the frequency of IL4 expression and IFNγ/IL10 responses in health further supports what Akdis and colleagues described as a “fine balance between Th1, Th2 and Tr1-like responses” [9].

We also confirm important differences between T cell responses to cat allergen and grass pollen allergens. Studies with MHC class II tetramers suggest a higher frequency of circulating T cells responding to perennial compared to seasonal allergens [5]: on the basis of the CD154 response, our data support this view, although the finding must be interpreted with caution as comparison is difficult when stimulating with different allergen extracts, even when the experiments are optimised. It has been hypothesised that responses to perennial allergens are directed at a broader range of epitopes compared to seasonal allergens, resulting in a larger composite signal [5,13,14].

Secondly, we demonstrate that the frequency of CD27- effector memory T cells within the Th2 compartment was significantly higher in grass-allergic participants, supporting the view that T cell responses to seasonal allergens comprise a higher proportion of late-differentiated T cells [5]. In contrast, Th2 cells in cat-allergic individuals were consistent with an early-differentiated phenotype. A number of groups have demonstrated a predominantly early-differentiated phenotype in T cells responding to cat allergen using MHC tetramer experiments [4-8]. A recent study by Wambre and colleagues also demonstrated early- and late-differentiated T cell phenotypes for perennial (house dust mite) and seasonal (birch) allergens [5]. It has been hypothesised that chronic allergen exposure could maintain a persistent central memory profile for perennial allergens. Wambre and colleagues investigated the differentiation stages of Aln g 1-specific T cells before and after specific immunotherapy for alder pollen allergy [6]. Pathogenic responses, defined as Th2 cytokine production from CRTh2^+^ cells, were specifically associated with the late-differentiated CD27- T cells, dominating in allergic individuals but absent in healthy control subjects. Specific immunotherapy induced depletion of this CD27- Th2 cell population in favour of a predominantly CD27^+^ “protective” T cell response, therefore indicating the expression of CD27 can define distinct subsets of allergen-specific T cells. Similarly, Bonvalet et al. illustrated a reduction in CD27- Th2 cells following grass pollen-specific immunotherapy, although this did not correlate with clinical efficacy [4]. Certainly, central memory T cells have been associated with persistent long-lived responses to antigens and in persistent viral infections. Literature investigating virus-specific lymphocytes hypothesised the nature and timing of antigen stimulation may influence the generation of memory T cells [15], in which chronic viral infection, such as cytomegalovirus, is associated with a central memory T cell phenotype [16]. In addition, CD27 expression has been utilised to discriminate active and latent tuberculosis infection, in which the loss of CD27 expression marks active infection, compared to latent, chronic infection exhibiting high CD27 expression [17].

Asymptomatic sensitisation – where IgE results are positive in the absence of a clinical history of symptoms upon exposure – is an important issue in clinic. An assay capable of discriminating the two would be highly desirable, particularly in the settings of food, drug and venom allergy. Unfortunately, the Th2:Th1 ratio and other T cell-based markers in this assay were not able to discriminate subjects with asymptomatic sensitisation and true clinical allergy to grass, birch and cat allergens. It is possible that more refined assays with a greater number of markers could have improved predictive value, or it may be that T cell responses are too upstream of early phase symptoms to be useful.

## Conclusions

We confirm our previous descriptions of a close relationship between Th1, Th2 and Tr1-like responses to grass and cat allergen in health that maintains a low Th2: Th1 ratio. In allergic individuals, this relationship is dysregulated with a higher Th2: Th1 ratio and IgE concentration that correlates with the frequency of Th2 cells. In keeping with tetramer-based studies, we illustrate a higher proportion of late-differentiated Th2 cells responding to seasonal allergens compared to the early-differentiated phenotype of cat-specific Th2 cells. The detection of CD154^+^ T cells following short-term allergen stimulation is a useful method for the investigation of allergen-specific T cells, but using our current panel does not discriminate between allergy and asymptomatic sensitisation.

## Methods

### Study participants

This study was approved by the National Research Ethics Service (South East Coast, Brighton and Hove) and the University of Sussex Ethics Research Committee. All volunteers provided informed written consent. Cat-allergic (n = 20) and grass-allergic (n = 19) participants were recruited from the Allergy Clinic of the Royal Sussex County Hospital (Table 1). All grass-allergic participants had a history of summer rhinitis and were SPT-positive to grass pollen extract. All cat-allergic participants had a history of cat-induced rhinitis and were SPT-positive to cat dander extract.

**Table 1:** Participant demographics

	**Non-allergic (Cat control)**	**Cat-allergic**	**Non-allergic (Grass control)**	**Grass-allergic**
**No. of participants**	18	20	18	19
**Age**	30 ± 8	34 ± 14	26 ± 7	39 ± 15
**Sex (M:F)**	12	5	10	8
	6	15	8	11
**Allergic diseases (%):**				
**Asthma**	0	50	0	47
**Atopic dermatitis**	0	25	0	42
**Rhinitis:**				
**Seasonal**	0	100	0	100
**Perennial**	0	40	0	47
**Cat-Induced**	0	100	0	68
**Pollen food syndrome**	0	40	0	63
**SPT-positive (%):**				
**Birch**	0	95	0	74
**Grass**	0	90	0	100
**Cat**	0	100	0	74

Two approximately age-matched non-allergic populations (n = 18) were recruited from staff and students at Brighton and Sussex Medical School: these participants had no history of atopic diseases and negative skin prick tests to common aeroallergens (birch pollen, grass pollen mix, early pollinating tree pollen mix, mid-pollinating tree mix, cat dander). Blood samples were taken a minimum of eight weeks outside the grass pollen season.

### Antigens

Birch pollen, grass pollen and cat dander allergen extracts was a kind gift of Dr Helga Kahlert (AllergoPharma, Reinbek, Germany). The freeze-dried lyophillisate was reconstituted with phosphate buffered saline (PBS) to 2×10^5^ protein nitrogen units/ml (PNU/ml) (1330 μg/ml) and filtered before storage at -20°C. The final LPS concentration in the stimulation system was estimated at 1.54 ng/ml by LAL test (Pyrochrome C180, Pyroquant Diagnostik, Germany). Phytohaemagglutinin (PHA) (Sigma Aldrich, Dorset, UK) was reconstituted in PBS to 1 mg/ml before storage at -20°C.

### Allergen stimulation

Human peripheral blood mononuclear cells (PBMC) were isolated from citrated blood by density centrifugation using Ficoll Paque Plus (GE Healthcare, Buckinghamshire, UK), washed twice in PBS then resuspended at 5×10^6^/ml in RPMI 1640 (10% FCS, 2 mM L-Glutamine, 1% Penicillin-Streptomycin). PBMC were then rested for 24 hours at 37°C and 5% humidified CO_2_ to improve allergen-induced CD154 expression [Smith]. After resting, 2×10^6^ PBMC were stimulated with allergen at 1000PNU/ml in a total volume of 500 μl, in 5 ml polypropylene tubes at 37°C and 5% humidified CO_2_ for 2 hours, before the addition of 20 μg/ml Brefeldin A in 500 μl (10 μg/ml final concentration, Sigma Aldrich, Dorset, UK) for the final 14 hours. Unstimulated PBMC served as a negative control for background correction. 10 μg/ml PHA (Sigma Aldrich, Dorset, UK) was used as a positive control.

### Antibody staining

Cells were washed in FACS buffer (PBS, 0.5% BSA, 0.1% sodium azide). Surface staining was performed by incubation for 30 minutes with various combinations of anti-human CD3-Alexafluor700, CD4-PerCP, CD27-FITC (BD Pharmingen, Oxford, UK), CD25-APC-Cy7 (Biolegend, Cambridge, UK), CD45RA-ECD (IQ Products, DL Groningen, The Netherlands), CD4-ECD (Beckman Coulter, High Wycombe, UK) and Aqvid dead cell stain (Invitrogen, Paisley, UK). Cells were then washed in FACS buffer, fixed and permeabilised (lyse and permeabilisation II solutions, BD Pharmingen). Intracellular staining was performed for 30 minutes with various combinations of anti-human IFNγ-PeCy7 (BD Pharmingen), CD154-PacificBlue, IL-4-PE and IL-10-APC (Biolegend). Fluorochrome-conjugated beads were used for compensation. Fluorescence-minus-one (FMO) controls were performed to ensure the compatibility of fluorochromes and to eliminate antibody effects in cytokine expression.

### Flow cytometric analysis

Cells were washed with FACS buffer prior to acquisition by LSR II flow cytometer (BD Biosciences, Oxford, UK) with FACSDiva. A minimum of 4×10^5^ CD4 events were gated for each subject. Aqvid staining demonstrated cell viability close to 100%, therefore live-dead exclusion was not routinely performed. Data were analysed using FlowJO software v9.9.3 (Treestar, Ashland, US). Boolean gating combinations were computed for cytokine and cell marker analysis.

### Cat-specific IgE

Serum samples were frozen at -80°C before batch analysis for cat dander IgE concentration, using a Phadia 100 instrument (Thermofisher IDD, Uppsala, Sweden).

### Statistical analysis

Statistical analysis was supported by Graphpad Prism v5.03 (La Jolla, USA). The data distribution was non-parametric according to the D’Agostino and Pearson omnibus normality test. Median values were used for comparison throughout. All cell frequency values were background-corrected by subtraction of the unstimulated cell frequency from the stimulated cell frequency. Statistical significance was calculated using the two-tailed Mann–Whitney *U* test with a significance level of 0.05. Spearman rank correlation analysis was used to investigate statistical dependence between variables. To calculate Th2: Th1 ratio, the frequency of CD154^+^IL-4^+^ cells was divided by the frequency of CD154^+^IFNγ^+^ cells. Where participants had no detectable CD154^+^IFNγ^+^ or CD154^+^IL-4^+^ cells, a value was defined by allocating a predicted frequency value based on the regression equation from all responding participants.

## Abbreviations

FMO: Fluorescence Minus One; MHC: Major Histocompatibility Complex; PBMC: Peripheral Blood Mononuclear Cells; PBS: Phosphate Buffered Saline; PHA: Phytohaemagglutinin; PNU: Protein Nitrogen Units; RPMI: Roswell Park Memorial Institute.

## Competing interests

The authors declare that they have no competing interests.

## Authors’ contributions

KS carried out the PBMC preparations, allergen stimulation and antibody experiments, flow cytometric analysis and helped to draft the manuscript. NG carried out the recruitment of participants and PBMC preparations. FS performed the serum cat- and grass-specific IgE experiments and analysis. EC conducted the statistical analysis. AF and FK provided technical assistance and contributed to study design, coordination and participant recruitment. MT conceived the study, coordinated the study design, experimental and statistical analysis and participant recruitment. All authors read and approved the final manuscript.
